# The Role of Medication Beliefs in COVID-19 Vaccine and Booster Uptake in Healthcare Workers: An Exploratory Study

**DOI:** 10.3390/healthcare11131967

**Published:** 2023-07-07

**Authors:** Carys Dale, Catherine Heidi Seage, Rhiannon Phillips, Delyth James

**Affiliations:** 1 School of Healthcare Sciences, College of Biomedical and Life Sciences, Cardiff University, Cardiff CF10 3AT, UK; 2 School of Sport and Health Sciences, Cardiff Metropolitan University, Cardiff CF5 2YB, UK; hseage@cardiffmet.ac.uk (C.H.S.); rphillips2@cardiffmet.ac.uk (R.P.); dhjames@cardiffmet.ac.uk (D.J.)

**Keywords:** COVID-19, vaccination, booster, healthcare workers, vaccine hesitancy, self-regulatory model, necessity–concerns framework, illness perceptions, vaccine delay, vaccine uptake

## Abstract

Illness and medication beliefs have shown to predict COVID-19 vaccination behaviour in the general population, but this relationship has yet to be demonstrated in healthcare staff. This research aimed to explore the potential explanatory value of illness and medication beliefs on the COVID-19 vaccination uptake of a sample of patient-facing healthcare workers (HCWs). A web-based questionnaire—measuring beliefs about vaccinations (the BMQ), perceptions of COVID-19 (the BIPQ), vaccine hesitancy, and vaccine uptake—was targeted to HCWs via social media platforms between May–July 2022. Open text responses allowed participants to provide explanations for any delay in vaccine uptake. A total of 91 participants completed the questionnaire. Most respondents (77.1%, *n* = 64) had received three doses of the COVID-19 vaccination, and vaccination uptake (number of doses received) was predicted by Vaccine Concerns, Vaccine Hesitancy, and their Necessity–Concerns Differential score. Vaccine Hesitancy was predicted by Necessity, Concerns, and Overuse scores, as well as Necessity–Concerns Differential scores. Delay in Vaccine Uptake could only be predicted for Dose 3 (Booster). Qualitative data revealed that hesitant respondents were “unable to take time off work” for vaccination and that some had concerns over vaccine safety. In conclusion, illness and medication beliefs have potential value in predicting vaccine hesitancy and uptake in healthcare workers. Interventions to improve vaccination uptake in this population should address concerns about vaccine safety and releasing staff for vaccination booster appointments should be prioritised. Future research should further investigate the relationship between illness and medication beliefs and COVID-19 vaccine uptake in a larger sample of healthcare workers.

## 1. Introduction

The World Health Organisation declared the outbreak of SARS-CoV-2 as a worldwide pandemic on 11 March 2020 [1], leading to a global response in the form of vaccination development. Following the approval of the Pfizer-BioNTech COVID-19 vaccine in December of 2020, vaccination uptake has been a crucial point of investigation when considering behaviour during the SARS-CoV-2 pandemic [2]. Current research suggests that vaccination against the COVID-19 disease reduces serious illness by at least 80% and reduces transmission in those that have received a full course of vaccinations [3]. However, in the UK, many individuals are still hesitant, or resistant, to having the COVID-19 vaccination, which at the time of data collection had been widely available for over a year [3]. Whilst at the time of writing coronavirus has a less significant impact than in recent years, it continues to circulate in communities on a long-term basis, bringing with it an ongoing challenge. It is important to gain understanding into vaccination behaviour in a pandemic context both in preparedness for any future waves or variants and possible future pandemics that may occur. In this way, the timing of data collection is notable—attitudes towards the vaccination are likely to have changed in the time elapsed between its initial release and a year afterwards, and therefore it is important to consider how perceptions may change as time goes on.

A core element of the worldwide strategy to reduce COVID-19 transmission was to ensure a high vaccination uptake rate within healthcare workers (HCWs). Many HCWs work with (or in very close proximity to) high-risk individuals, and therefore vaccination should be encouraged in the maximum number of HCWs to reduce the risk of transmission to immunocompromised, or otherwise high-risk, patients in hospital care. Due to these increased risks, healthcare workers in the UK were amongst the first population groups to be invited to receive the vaccination at rollout [4,5]. Whilst COVID-19 vaccine uptake has been higher in HCWs than in the general population, UK data published in 2022 suggests that around 10% of HCWs were not fully vaccinated [5]. As the vaccination of HCWs is imperative to herd immunity efforts, understanding why some HCWs may delay uptake of the vaccination will provide valuable knowledge to contribute to vaccine uptake interventions for this population [4,6].

In order to understand the psychological influences on vaccination uptake, hesitancy behaviours can be understood through the lens of the Self-Regulation Model of Illness (SRMI) [7]. The SRMI explains that individuals seek to understand an illness (such as COVID-19) by developing an illness representation, informed by personal experience, information gathered and the behaviour of those around them. As measured by the Brief Illness Perception Questionnaire [8], perceptions of identity, cause, consequences, timeframe, personal control, treatment control, concerns, emotions, and illness comprehensibility will determine the perceived threat of the illness. As most HCWs worked throughout the COVID-19 pandemic, it is likely that their personal experience of the illness influenced their perceived risk of infection and transmission than the general public [9]. HCWs are also exposed to regulated and reliable sources of information within healthcare settings about the illness [10]. Included in the SRMI is the Necessity–Concerns Framework (NCF), which is key to understanding vaccine decision making. The NCF describes how individuals assess the need for a medication (in this case, a vaccination) against their concerns about the medication. The framework postulates that, in forming an attitude towards a medication, an individual will consider how necessary they believe it to be (for example, for their own health or for the protection of others) and how concerned they feel about taking the medication (for example, due to potential negative side effects). Whether or not the individual chooses to take the medication will depend on which of these factors they give more weighting [10]. These beliefs can be measured using the Beliefs about Medicines Questionnaire [11], a psychometrically robust measure which captures an individual’s beliefs about medicines prescribed specifically to the individual, and towards medicines in general. This theoretical model [7] suggests that vaccination behaviour is influenced by constructs such as perceived threat and personal exposure to illness, as well as the consideration of vaccine necessity. One factor that may be influential is perceived professional obligation. In findings from qualitative research of HCW attitudes towards the influenza vaccination, themes of obligation were identified, with many individuals reporting that they felt obliged to receive the vaccine for the safety of their patients [12]. This may influence the likelihood of an HCW receiving COVID-19 vaccinations and could make them less hesitant to receiving the vaccination in comparison to non HCWs.

Research investigating COVID-19 vaccination behaviour in the general population has revealed a relationship between the NCF and vaccine acceptance—in a study conducted during the initial stages of vaccination efforts, the NCF was used as a theoretical model to predict vaccine uptake in a sample of the general public in Portugal [13]. It was found that greater perceptions of necessity concerning the vaccine (for personal and global health reasons) predicted a higher likelihood to receive the vaccine, whereas greater levels of concern over vaccine safety (often due to a perceived lack of scientific evidence basis) led to reduced likelihood of receiving it [13].

In addition to this, research conducted during the initial stages of the pandemic has indicated that the NCF may be a useful explanatory framework for HCW vaccination behaviour. Research investigating HCW vaccination behaviour through the theoretical domains framework (TDF) has identified a trend in higher perceived benefits of vaccination relating to a greater willingness to be vaccinated [14]. In addition to this, a review of the global literature surrounding COVID-19 vaccine behaviour in HCWs indicated that beliefs about consequences (such as side effects and long-term health affects) were key in predicting vaccine acceptance [15]. In the UK specifically, HCWs cited fears over long-term side effects as reasoning for vaccine hesitancy—however, this did not necessarily deter them from receiving the vaccine [16]. It is worth noting that, whilst ongoing research is investigating long-term side effects of receiving a COVID-19 vaccination, experiencing long-term effects is uncommon [17]. Research has also shown that a perceived lack of necessity—for example, through a perceived low susceptibility to infection—has influenced vaccination decision making in HCWs [18]. It is clear in the literature that elements of the NCF, including the consideration of perceived necessity (through perceived professional obligation and health protection) and concerns (surrounding side effects and perceived lack of evidence for vaccine safety) have a significantly influential impact on whether HCWs choose to receive vaccinations. However, the NCF has yet to be utilised as an explanatory framework in a sample of HCWs.

Furthermore, little research has yet been undertaken to investigate HCWs’ attitudes towards booster vaccinations as we enter the later stages of the pandemic. Initial research in a sample of Canadian health professionals suggested that booster doses were less trusted and seen as less necessary in those studied [19]. In addition to this, vaccine uptake data in Wales, UK show that fewer HCWs are receiving boosters in comparison to those who received the first two doses, suggesting that the perceptions of necessity that encouraged initial vaccination are less influential when considering the booster doses [20].

Current research suggests that many HCWs consider the benefits and drawbacks of receiving the COVID-19 vaccination when deciding whether to receive it [14,15,19]. However, the NCF has yet to have been employed as a theoretical model to predict vaccination behaviour in this population, and the influence of this on booster uptake has not yet been investigated during the latter stages of the pandemic. Therefore, the aim of the present study was to explore how the Necessity–Concerns Framework influences COVID-19 vaccination and booster uptake in HCWs and to pilot the use of modified versions of the BMQ and BIPQ to measure this.

### This Exploratory Study Had Two Objectives

(1) To explore the role that the SRMI and NCF may have on COVID-19 vaccination and booster uptake in a sample of HCWs.
(2) To investigate the reasoning for delay in vaccination update through open-ended free text responses.

## 2. Materials and Methods

Ethical approval was gained from Cardiff Metropolitan University, School of Sport and Health Sciences, Applied Psychology Ethics Committee (Reference number PGT-5752).

An online, mixed-methods questionnaire was prepared using Qualtrics (https://www.qualtrics.com) and distributed to healthcare workers. This questionnaire investigated beliefs about vaccinations in general, beliefs about COVID-19 vaccinations specifically, illness perceptions towards COVID-19, vaccine hesitancy, and delay in vaccination uptake. A mixed methods approach through quantitative questionnaires and free-text responses was adopted to provide reasoning for vaccine refusal or delay, therefore providing an explanation for quantitative findings according to Bryman’s scheme of mixed-methods research justification [21].

### 2.1. Participant Recruitment

Participants were recruited by voluntary recruitment. Information was shared onto Twitter™ and Facebook™ through the personal profiles of the investigators, as well as to targeted Facebook groups for different subgroups of HCWs to increase diversity in the sample. Social media recruitment was utilised to maximise the number of participants that could be recruited, as social media recruitment has been shown to lead to improved enrolment in comparison to other recruitment methods [22]. Respondents were encouraged to share the online link with other eligible potential participants. There was a six-week data collection period in which the survey link was active from May–July of 2022. Individuals were eligible for participation if they were over 18 years of age, were currently employed in a patient-facing role in a healthcare setting (such as nurses, doctors, healthcare assistants, care assistants, pharmacists) and were employed in a healthcare setting in the UK during the COVID-19 pandemic (March 2020–Present). Individuals were excluded from participation if they did not work in a healthcare role during the pandemic (March 2020–Present), if they worked in healthcare settings but did not have a patient-facing role (for example, administrative workers in care homes), and if they lived and worked outside of the UK.

### 2.2. Measures

Beliefs about Medicines Questionnaire—[11] this consists of two separate measures: the BMQ Specific (modified for the COVID-19 vaccine), which measured beliefs about the COVID-19 vaccine, and the BMQ General (modified for vaccinations in general), which assessed beliefs about vaccinations in general [11].

The Brief Illness Perception Questionnaire (BIPQ) [8] comprises five scales and is based upon the eight components of illness perception described by Leventhal’s Self-Regulation Model of Illness [7]. These five scales assess identity, cause, timeline, consequences, cure/control, concern, emotions, and illness comprehension and were modified to be specific to COVID-19. This measure was selected as it has been previously utilised to measure the perceived threat of COVID-19 [23].

The Oxford COVID-19 Vaccine Hesitancy Scale [24] is a seven-question scale originally designed to assess hesitancy towards a potential COVID-19 vaccine before a vaccine was made available to the public. The scale contains Likert scale questions including ‘*I would describe my attitude towards receiving a COVID-19 vaccine as*’ with possible responses ranging from ‘Very Keen’ to ‘Against It’. For the purposes of the present study, questions were modified to assess current attitudes to the existing COVID-19 vaccine. Individual items were scored from 1 to 5, with one indicating low levels of hesitancy and five indicating high levels of hesitancy.

### 2.3. Outcome Variables

A bespoke scale was developed to measure *Vaccine Uptake and Delay to Vaccine Uptake.* This scale required participants to reveal how many doses of the vaccination they had currently received, state when their initial invitation for each dose was received, and how many weeks following this invitation the vaccine dose was administered. The number of weeks taken to receive vaccination was used as a measure of Vaccination Delay. Space was provided for participants to explain for what reason, if any, they delayed receiving the vaccination.

Information regarding age, ethnicity, geographic location, gender, and healthcare role was also collected. 

### 2.4. Data Analysis

Statistical analysis was conducted using IMB Statistical Package for The Social Sciences (SPSS; Version 27) (IBM, New York, NY, USA) [25].

Scores for each construct of the BMQ (Vaccine Necessity, Vaccine Concerns, Vaccine Overuse, and Vaccine Harm) were calculated by summing the individual scores for each question pertaining to that construct, with higher scores indicating a higher level of agreement. Necessity–Concerns Differential scores were calculated for each participant by subtracting the Concerns score from the Necessity score to give a measure of the difference between the two outcomes.

BIPQ scores were calculated for each participant as a measure of perceived threat of COVID-19. This score was computed by summing the scores from each item with items 1, 2, 5, 6, and 8 being scored as 0 = 1 points, 10 = 11 points, and items 3, 4, and 7 being reverse scored as 0 = 11 points and 10 = 1 point to give a total score out of 88. The higher the score on the BIPQ, the greater threat the participant considers an illness to have over their individual health. This approach to the analysis of BIPQ scores is often adopted in the literature [23].

For the Oxford Hesitancy Scale, total scores for each participant were calculated by summing the scores for each item, with higher scores indicating higher levels of hesitancy. A total score out of 35 was given for each participant.

Multiple linear regression was used for analysis of the variables. As the aim of this study was exploratory, statistical rules of thumb suggests that 2 subjects per variable (SPV) is acceptable for regression analysis and was therefore an appropriate statistical method for this sample (with 8 SPV) [26]. Linear regression analysis tested the predictive value of scores on the two scales of the BMQ General and BMQ Specific (Necessity–Concerns and Overuse–Harm), Necessity–Concerns Differential, and scores on the Brief-IPQ and Vaccine Hesitancy measures on Vaccine Delay (as determined by scores on the Weeks Taken to Accept Vaccination). If the alternate hypothesis was to be accepted, a significant proportion of the variance in vaccine delay would be accounted for by the model.

Linear regression analysis was also used to identify if Vaccine Hesitancy and Delay in Vaccine Uptake could be predicted by the other variables used in the model. Logistic regression analysis tested whether the independent variables could predict whether individuals delayed or did not delay receiving vaccinations. Descriptive statistics were also conducted to test the influence of age, gender, profession, ethnicity, and location on Vaccine Delay and Vaccine Hesitancy.

## 3. Results

A sample of 124 individuals responded to the online invitation to participate and started the questionnaire. The completion rate was 73.3% (*n* = 91), with 16 participants (12.9%) not completing the Vaccine Delay question and 17 participants (13.7%) not completing other parts of the survey. This response rate is comparable to existing research of a similar nature [27]. Of the completed responses, nine (7.0%) were excluded from analysis for the following reasons: seven (5.6%) did not work in patient-facing roles, and two (1.6%) did not work in the UK. The remaining sample of 82 responses was utilised in analysis. Logistic regression analyses revealed that completing/not completing the questionnaire was not predicted by the independent variables in the model (X2 = 4.590, d.f. = 6, *p* = 0.597).

Demographic information from the sample used in analysis can be found in Supplementary Material S1. The most frequently reported age category was 46–55 years (39.1%, *n* = 32), ethnicity was White British (86.6%, *n* = 71), profession was nurse (48.7%, *n* = 40), and location was Channel Islands (56.1%, *n* = 46).

### 3.1. Distribution of Scores in the Independent Variables

Whilst scores for BMQ-Necessity, BMQ-Concerns, BMQ-Harm, and BIPQ scales were normally distributed, scores for BMQ-Overuse (Vaccines) and Vaccine Hesitancy had a positive skew, meaning lower scores were more common for these measures. The distribution of Necessity–Concerns Differential scores had a negative skew, meaning higher scores (indicating higher perceived necessity than concern) were more common. Descriptive statistics and Cronbach’s alpha scores for the Independent Variables in the model can be found in Supplementary Material S2.

### 3.2. Vaccination and Booster Uptake

**Figure 1:**
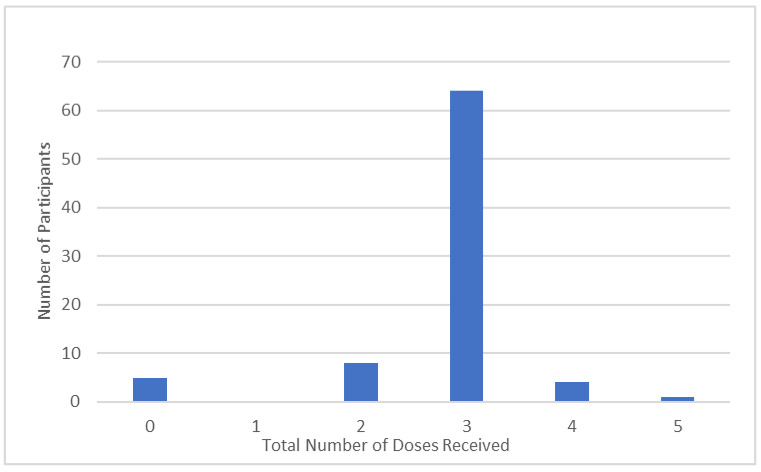
Number of participants reporting having received each dose of the COVID-19 Vaccination (*n* = 82).

The majority of participants reported having received three doses of the COVID-19 vaccination (77.1%, *n* = 64). A smaller percentage reported having received two doses (9.8%, *n* = 8), four doses (4.9%, *n* = 4), and five doses (1.2%, *n* = 1). A percentage of 6.1% (*n* = 5) reported that they had not received any doses of the COVID-19 vaccination. No respondents reported receiving only one dose of the vaccination (Figure 1).

Vaccine Hesitancy and Vaccine Uptake were found to be significantly correlated, with higher hesitancy relating to fewer doses received (r^82^ = −0.669, *p* < 0.001, Supplementary Material S3).

### 3.3. Predictive Power of the NCF

A multiple linear regression was performed to assess the predictive power of BMQ-Harm (Vaccines) BMQ-Overuse (Vaccines), BMQ-Concern, BMQ-Necessity, Necessity–Concerns Differentials, Vaccine Hesitancy, and BIPQ scores on Vaccine Uptake (Number of doses received). The model was found to be significantly predictive of Vaccine Uptake (F^6,76^ = 12.034), accounting for 45% of the variance (adj𝑅^2^ = 0.450) (Table 1).

BMQ-Concerns scores (Beta = 0.242, t^76^ = 2.017, *p* = 0.047) and Vaccine Hesitancy scores (Beta = −0.666, t^76^ = −3.874, *p* < 0.001) were significantly predictive of Vaccine Uptake. Necessity–Concerns Differentials were also predictive of Vaccine Uptake, (Beta = 0.509, t^76^ = 5.283, *p* < 0.001).

#### 3.3.1. Vaccine Delay

A minority of the participants who had accepted a dose of the vaccine reported a delay in receiving Dose 1 (30.1%, *n* = 22), Dose 2 (28.9%, *n* = 23), and Dose 3 (36.1%, *n* = 25). For the fourth dose, 50% (*n* = 2) reported a delay; however, only four individuals reported that they had received four doses. (Table 2).

A multiple linear regression performed to assess the predictive power of BMQ-Harm (Vaccines), BMQ-Overuse (Vaccines), BMQ-Concern, BMQ-Necessity, Necessity–Concerns Differentials, BIPQ, and Vaccine Hesitancy scores on Vaccine Delay for Dose 1 was found not to be significantly predictive (F^6,76^ = 0.703, *p* = 0.648), accounting for 2.4% of the variance (adj 𝑅^2^ = −0.024). A multiple linear regression was also performed to assess the predictive power of the model for Dose 2. The model was not a significant predictor of Vaccine Delay for Dose 2 (F^6,76^ = 1.031, *p* = 0.413), accounting for 0.2% of the variance (adj 𝑅^2^ = −0.002).

#### 3.3.2. Dose 3

A multiple linear regression was performed to assess the predictive power of the model on Vaccine Delay for Dose 3.

The variables in the model significantly predicted Vaccine Delay for Dose 3 (F^6,76^ = 3.033, *p* = 0.011), accounting for 15% of the variance (adj 𝑅^2^ = −0.150, Table 3).

BMQ-Overuse (Vaccines) scores were significantly predictive of Vaccine Delay for Dose 3 (Beta = −0.546 t^76^ = −3.123, *p* = 0.003), as were Vaccine Hesitancy scores (Beta = 0.557 t^76^ = 3.080, *p* = 0.003).

Regression analyses were not performed for Dose 4 or 5 due to the limited number of participants reporting having received fourth or fifth doses.

#### 3.3.3. Predictive Value for Delay/No Delay

A bivariate logistic regression was performed to assess the power of the model in predicting whether individuals delayed or did not delay uptakes for all doses. The model was not a significant contribution to predicting Vaccine Delay.

The logistic regression was also run with delays unrelated to hesitancy (such as being positive for COVID-19 at time of appointment) removed from the analysis, but the regression remained non-significant.

### 3.4. Vaccine Hesitancy

A multiple linear regression was performed to assess the predictive power of BMQ-Harm (Vaccines), BMQ-Overuse (Vaccines), BMQ-Concern, BMQ-Necessity, Necessity–Concerns Differentials, and BIPQ scores on Vaccine Hesitancy. The model was found to be significantly predictive of Hesitancy scores (F^6,76^ = 50.894), accounting for 75.5% of the variance (adj𝑅^2^ = −0.755) (Table 4).

Further study into these results revealed that BMQ-Necessity (Beta = −0.437, t^76^ = −5.258, *p* < 0.001) BMQ-Concerns (Beta = 0.226 t^76^ = 2.995, *p* = 0.004), and BMQ-Overuse (Vaccines; Beta = 0.306, t^76^ = 3.071, *p* = 0.003) scores were significantly predictive of Vaccine Hesitancy. Necessity–Concerns Differentials were also predictive of Vaccine Hesitancy, (Beta = −0.834, t^76^ = −13.752, *p* < 0.001).

Table 5 shows the percentage of the sample that are fully vaccinated (three or more doses received) categorised by occupation.

### 3.5. Reasoning for Vaccination Delay

Participants were given the option to explain what reason, if any, they had for delaying receiving the vaccination. Qualitative analysis was not performed on explanation statements due to the limited quantity of these data. Whilst only 19 out of the 30 participants who reported a delay chose to give a reason, this open-ended question provided valuable data. Out of the 19 respondents who gave reasoning, eight reported “*being positive for COVID-19*” as the reason for delay. “*Short Staffed/Couldn’t get time off work*” was reported by three individuals. Pregnancy was cited as a reason for delay for three individuals, with two stating they were “*unsure whether it was safe during pregnancy*”, and one participant stating that they “*only took the vaccine whilst pregnant*” and so delayed later doses. “*Received vaccine for travel*” was cited by one participant, with the remaining three participants giving hesitancy as reasoning, claiming “*I didn’t think it was necessary*” and “*Anti-vaxx friends were causing doubt*”.

#### Summary

In summary, Vaccine Hesitancy could be predicted by perceptions of necessity, vaccine concerns, beliefs about vaccine overuse, and the Necessity–Concerns Differential. Vaccine Hesitancy and Vaccine Uptake were significantly related, with Vaccine Uptake being significantly predicted by Vaccine Hesitancy, vaccine concerns, and Necessity–Concerns Differential scores. The model was only significantly predictive of Vaccine Delay for Dose 3, with beliefs about vaccine overuse and Vaccine Hesitancy being the most significant predictors. These results suggest that whilst the predictors in the statistical model are significantly related to both hesitancy and doses received, this did not significantly influence weeks delay in receiving the vaccine until the third dose.

## 4. Discussion

This cross-sectional research was conducted to explore the explanatory power of the Necessity–Concerns Framework [23] (NCF) on the COVID-19 vaccination and booster uptake in a sample of healthcare workers (HCW). A web-based survey was developed to pilot modified versions of the Beliefs about Medicines Questionnaire [11], Brief Illness Perception Questionnaire [8], and Oxford Vaccine Hesitancy Scale [24], along with the novel scale which was developed to measure Vaccine Delay. The results of this study can be used to inform the designing and conducting of interventions to encourage COVID-19 vaccine uptake in healthcare staff, as well as validating the use of the modified measures developed.

Over two-thirds of respondents had received three or more doses of the coronavirus vaccine, and most reported no delay. Very few (6%, *n* = 5) respondents had refused vaccination, which is consistent with UK Government data on vaccine uptake in HCWs [5]. Vaccine Uptake was significantly predicted by the theoretical model, with BMQ-Concerns, Necessity–Concerns Differentials, and Vaccine Hesitancy being the significant predictors. In addition to this, Vaccine Hesitancy was significantly predicted by the Necessity–Concerns Differential, perceptions of necessity and concerns independently, and perceived vaccine overuse. The independent variables were only able to predict Vaccine Delay for Dose 3, the ‘booster’ dose. Moreover, uptake for the booster dose was slightly reduced in this sample (around 7% fewer participants received booster doses in comparison to first and second doses). A recent survey of booster vaccinations in Welsh HCWs found a drop off in vaccine uptake for the third dose, suggesting that the findings in the small sample used in the present study reflect behaviours in the wider population [20]. There are several potential explanations for this finding—the third ‘booster’ dose of the COVID-19 vaccine was released for HCWs in September of 2021, six months after this group were invited to receive their second dose [5,28]. Over time, hesitancy may have increased, especially if the third dose was perceived as less necessary than the original course, as was suggested by HCWs in previous research [19]. Research has also shown that uptake of the booster dose was reduced in those who experienced negative side effects following receipt of the initial doses, which may have resulted in a dip in uptake in this sample [20].

It should also be noted that higher hesitancy scores did not necessarily predict lack of uptake or delay for every participant. This result has also been seen in previous research, indicating that HCWs may put perceived obligation above personal hesitancy when considering vaccination [16]. Therefore, the results of this paper show that hesitancy should be assessed independently of vaccine uptake, as in this population being hesitant did not necessarily result in an individual refusing vaccination.

The Necessity–Concerns Differential was predictive of Vaccine Uptake and Vaccine Hesitancy. This is in line with wider literature investigating COVID-19 vaccine willingness and suggests that the Necessity–Concerns Framework can be useful for understanding vaccine uptake behaviour [4,29]. Specific concerns about the COVID-19 vaccination being predictive of hesitancy is consistent with literature investigating the COVID-19 vaccine specifically, but less consistent with research concerning other, more common vaccinations. In previous research, concerns about safety were previously not significantly related to influenza vaccination intentions in HCWs, suggesting that the influence of specific concerns may be exclusive to the COVID-19 vaccination [30]. This is likely due to the novelty of the COVID-19 vaccination, as HCWs have reported higher concerns about vaccine safety for newly developed vaccinations [31]. BIPQ scores (as a representation of illness perceptions) were not significantly predictive of Vaccine Uptake, Vaccine Hesitancy, or Vaccine Delay. This is opposed to research in the general population [32], where a recent study found that illness perceptions correlated with COVID-19 vaccine intentions. This could suggest that illness perceptions are not predictive of vaccine behaviour in this sample; however, qualitative findings suggested that illness perceptions did, in fact, contribute to vaccine behaviour, as will be discussed below.

As there were few responses to the qualitative questions included in the survey, data of this nature were limited. Reasons related to actual hesitancy were only given in half of the explanation statements. The most reported reason for delay was “being positive for COVID-19”, suggesting that respondents were following advice from GOV UK [3], which advises waiting 4 weeks to receive vaccination following a positive COVID-19 result. Another commonly reported explanation was “not being able to take time off to receive the vaccine”. This highlights a key issue when encouraging vaccination in HCWs; staff members may be accepting of the vaccine and willing to receive doses, but unable to attend appointments due to staffing issues. This has important practical applications for how healthcare workplaces support staff members who are willing to become vaccinated. Further statements included hesitancy during or after pregnancy, and doubt seeded by vaccine-resistant friends. SRMI is key to explaining the impact of friends and family; information given by those close to us will have a stronger effect on illness representations, which in turn may then cause a delay in uptake [33]. This suggests that despite no significant statistical relationship found (potentially due to the limited sample or constructs not captured by the measures used), illness representations may have had influence on the delay in receiving the COVID-19 vaccination that was not captured by the measures, potentially due to the limited sample.

The results of this research have both psychological and practical implications. Primarily, the results suggest that the SRMI and NCF may be useful in to explaining vaccination behaviour in HCWs. The findings differed from previous findings regarding other, more well-known vaccinations, suggesting a difference in HCW attitudes towards the COVID-19 vaccination specifically. Whilst overuse, concern, and necessity scores were all predictive of self-reported hesitancy, this only influenced vaccine delay for Dose 3. This suggests that individual beliefs about vaccinations may significantly affect vaccine intentions for future doses, especially if the trend in diminishing uptake is continued. In order to encourage continued adherence to vaccination programmes, interventions intended for healthcare staff should focus upon addressing concerns about, and reemphasizing the necessity of, the vaccination itself, along with identifying any maladaptive beliefs about the overuse of vaccinations in general. Moreover, this research identified a barrier to receiving vaccinations in that staff could not take the necessary time from work to attend appointments. As such, healthcare workplaces should take every step to ensure those who wish to be vaccinated can receive doses by having on-site vaccination available. Previous research on interventions to improve influenza vaccine coverage in HCWs has found that on-site vaccination availability significantly improved vaccination uptake [34,35].

There are several limitations to the present study that must be considered when interpreting the results. Firstly, the sample under study was small, with 124 responses collected and 82 completed surveys used for analysis. This may have influenced both the power of any significant results and reduced the generalisability of the findings. However, as the purpose of this research was to explore the influence of a potentially useful theoretical model that was identified in the literature, the small sample employed in this research allowed for evidence to be developed that justifies further investigation of this relationship [15,16]. The use of an online survey design in this research has limitations in that it is difficult to guarantee the validity and accuracy of the data provided from individuals when recruiting through social media. This limitation is a common critique of online research, and this design was selected by the researchers to encourage a larger number of participants to participate in the survey [22]. In addition to this, there was an element of sampling bias that may have influenced results. Due to the nature of snowball sampling, the survey was primarily shared to HCWs living and working in the Channel Islands. As a result, 56.1% of respondents selected this category in the demographics section of the survey. This may result in it being challenging to translate these results to other devolved nations and further worldwide. However, vaccination rollout in the Channel Islands was similar to that in the UK [36]. HCWs in the Channel Islands were also likely experiencing similar external pressures and environments that influenced vaccine uptake, with the Channel Islands experiencing similar levels of infection and implementing similar health protection measures as the UK [37,38]. Therefore, the attitudes and experiences of the HCWs in this sample are likely to resemble those of HCWs in the rest of Britain. 

It is important to consider the point of data collection within the timeline of the COVID-19 pandemic. Data collection took place between May and July of 2022, over a year after vaccinations became available for frontline HCWs [3] and two years since COVID-19 was defined as a pandemic by the World Health Organisation [39]. As a result, the present research was predominantly retroactive, with respondents answering questions about their perceptions of a vaccine they had often already received. Illness representations of COVID-19 may have changed after two years of experience with the disease—the perceived threat of COVID-19 is a dynamic process that is often influenced by the current number of cases [35]. However, this also contributes to the novelty of the present study, as there is little research currently available that describes how vaccination attitudes, and attitudes towards the booster specifically, may have changed as the pandemic goes on. Whilst the pandemic is now towards its latter stages, the impact of living with COVID-19 in the community is still ongoing, and research investigating vaccine behaviour, particularly towards booster doses, remains to be valuable in the context of preparing health services for potential future waves, or future pandemics. Whilst the World Health Organization considers the pandemic to be over, it has been stressed that the impact of COVID-19 is far from over and that the risk of new variants emerging still exists, and therefore it must be ensured that we “learn from our mistakes” and prepare for the future [40].

Despite the given limitations, the value of the present study is supported by several methodological factors. The rigor of the present research was improved by pilot testing the survey used before its release, meaning that potential issues with the questionnaire could be identified and modified, increasing the validity of the survey used. This study allowed for the testing of novel measures. The BMQ Specific and General were modified to be applicable to COVID-19 vaccinations, and vaccinations in general, respectively. The BIPQ was adapted to be specific to COVID-19, and the Oxford Vaccine Hesitancy Scale was adapted to be useable after vaccinations became widely available. In addition to this, a novel measure of vaccine delay was developed and trialled as a part of this paper. Reliability calculations were performed on each of these adapted measures, with acceptable Cronbach’s alpha scores being attributed to each [41]. The testing of these measures allowed for their reliability to be assured, and, thus, their potential to be used in future research.

Further research should expand upon these findings and measure the predictive relationship of the NCF in a larger, more representative sample. This further research should build upon the qualitative findings of the present study to investigate in additional detail why some healthcare workers choose to delay vaccine uptake. Moreover, the modified measures of illness and medication beliefs should be tested on a sample of non-healthcare workers, allowing for comparisons to be made on how illness and medication beliefs in this population differ in their influence on vaccine uptake.

## 5. Conclusions

In conclusion, the Self-Regulation Model of Illness [33] and the Necessity–Concerns Framework [21] could be valuable theoretical tools for predicting Vaccine Hesitancy and Delay in Healthcare Workers. The Necessity–Concerns Differential can be used to predict both vaccine hesitancy and vaccine uptake, suggesting that the cost–benefit analysis suggested by Horne [25] significantly contributes to vaccine decision making in this population. The model was only significantly predictive of Vaccine Delay for the third dose, which suggests that in this sample, hesitancy only influenced vaccine uptake when it came to the ‘booster’ dose, potentially due to reduced perceptions of necessity. Importantly, in this sample, vaccine hesitancy did not necessarily result in delay or refusal. The results of this paper suggest that COVID-19 vaccine uptake interventions designed for healthcare workers should focus upon emphasizing the necessity of COVID-19 vaccinations, educating HCWs about the safety of such vaccinations, and addressing the concerns expressed. In this way, Hesitancy and Delay can be reduced and the maximum number of doses can be accepted by this critically important population.

## Figures and Tables

**Table 1:** Coefficients of multiple linear regression to assess the predictive power of the independent variables on Vaccine Uptake.

	Unstandardised Coefficients		Standardised Coefficients			Collinearity Statistics	
	B	S.E.	Beta	t	Sig.	Tolerance	VIF
BMQ-Necessity	−0.007	0.026	−0.038	−0.263	0.763	0.322	3.109
BMQ-Concerns	0.054	0.027	0.242	2.017	0.047 *	0.473	2.113
BMQ-Harm (Vaccines)	−0.047	0.044	−0.156	−1.066	0.290	0.318	3.147
BMQ-Overuse (Vaccines)	−0.019	0.037	−0.081	−0.508	0.613	0.271	3.697
Hesitancy	−0.079	0.020	0.666	−3.874	<0.001 **	0.230	4.348
BIPQ	0.003	0.008	0.037	0.356	0.723	0.643	1.554
NCD	0.060	0.011	0.509	5.283	<0.001 **	1.000	1.000

Note: Significance values with an single asterisk * indicate a *p* value of >0.05. Significance values with two asterisks ** indicate a *p* value of ≥0.001.

**Table 2:** Descriptive statistics of reported Vaccine Delay in delay following appointment in weeks (*n* = 82).

Delay (in Weeks)	n	Mean Avg.	S.E.	Min.	Max.
Dose 1	77	0.97	0.270	0	12
Dose 2	77	1.05	2.595	0	12
Dose 3	70	1.99	5.879	0	36
Dose 4	4	4.67	5.317	0	12

**Table 3:** Coefficients of multiple linear regression to assess the predictive power of the independent variables on Vaccine Delay for Dose 3.

	Unstandardised Coefficients		Standardised Coefficients			Collinearity Statistics	
	B	S.E.	Beta	t	Sig.	Tolerance	VIF
BMQ-Necessity	0.178	0.237	0.120	0.752	0.455	0.482	2.074
BMQ-Concerns	0.167	0.262	0.100	0.637	0.527	0.503	1.988
BMQ-Harm (Vaccines)	0.239	0.416	0.096	0.576	0.567	0.444	2.251
BMQ-Overuse (Vaccines)	−1.177	0.377	−0.546	−3.123	0.003 *	0.402	2.485
BIPQ	0.003	0.078	0.005	0.035	0.973	0.602	1.661
Hesitancy	0.680	0.221	0.557	3.080	0.003 *	0.377	2.655
NCD	−0.129	0.119	−0.130	−1.082	0.283	1.000	1.000

Note: Significance values with an single asterisk * indicate a *p* value of >0.05.

**Table 4:** Coefficients of multiple linear regression to assess the predictive power of the independent variables on Vaccine Hesitancy.

	Unstandardised Coefficients		Standardised Coefficients			Collinearity Statistics	
	B	S.E.	Beta	t	Sig.	Tolerance	VIF
BMQ-Necessity	−0.659	0.125	−0.437	−5.261	<0.001 **	0.439	2.280
BMQ-Concerns	0.423	0.141	0.226	2.990	0.004 *	0.529	1.890
BMQ-Harm (Vaccines)	0.138	0.247	0.055	0.560	0.577	0.319	3.134
BMQ-Overuse (Vaccines)	0.608	0.198	0.306	3.070	0.003 *	0.304	3.289
BIPQ	−0.047	0.048	−0.072	−1.050	0.296	0.653	1.532
NCD	−0.834	0.061	−0.838	−13.74	<0.001 **	1.000	1.000

Note: Significance values with an single asterisk * indicate a *p* value of >0.05. Significance values with two asterisks ** indicate a *p* value of ≥0.001.

**Table 5:** Vaccination behaviour in the sample categorised by profession.

Profession	N = (Percentage)	Percentage Fully Vaccinated
Nurse	40 (48.7)	92.5%
Operating Department Practitioner	11 (13.41)	90%
Healthcare Assistant	10 (12.2)	40%
Pharmacist	8 (9.75)	100%
Doctor	7 (8.53)	75%
Care Worker	3 (3.65)	87.5%
Pharmacy Technician	2 (2.43)	50%

## Data Availability

The data presented in this study are available on request from the corresponding author.

## Supplementary Materials

The following supporting information can be downloaded at: https://www.mdpi.com/article/10.3390/healthcare11131967/s1, Supplementary Material S1—Table of questions relevant to each construct of the BMQ, Supplementary Material S2—The relationship between Vaccine Uptake and Vaccine Hesitancy, Supplementary Material S3: The Relationship between Age and Vaccine Hesitancy, Supplementary Material S4—Demographic Variables in the Sample, Supplementary Material S4: Distribution of Scores in the Independent Variables, Supplementary Material S5: Coefficients of predictors in the model from the Logistic Regression, Supplementary Material S6: Quotations from Free-Text Responses, Supplementary Material S7: Quantitative Questionnaire (Exported from www.qualtrics.com).
