# Supplementary material for: The Role of Medication Beliefs in COVID-19 Vaccine and Booster Uptake in Healthcare Workers: An Exploratory Study
Source: Healthcare (Basel). 2023 Jul 7;11(13):1967. doi: 10.3390/healthcare11131967 (PMC10340697; doi:10.3390/healthcare11131967)
Supplement: Supplementary file 1 [file healthcare-11-01967-s001.zip › healthcare-2443875-supplementary.pdf]

Supplementary Material S1 – Table of questions relevant to each construct of the BMQ

Table 1: Relevant questions for each construct of the BMQ[25]

Measure	Construct	Total Score	Relevant Questions
BMQ-Specific	Specific Concerns	25	2, 5, 6, 8 & 9
	Specific Necessity	25	1, 2, 4, 7 & 10
BMQ-General	General Overuse	20	1, 3, 7, & 8
	General Harm	20	2, 4, 5, & 6

Supplementary Material S2 – The relationship between Vaccine Uptake and Vaccine Hesitancy

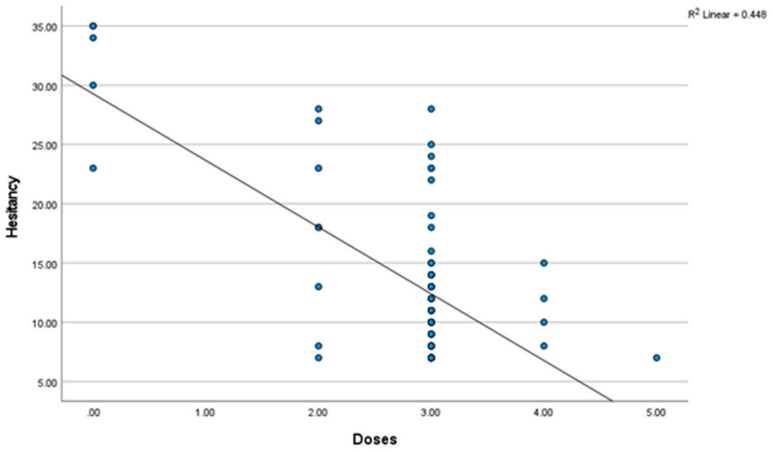

Figure 1. Scatter Plot showing the correlation between Vaccine Hesitancy and Vaccine Uptake (n=82)

Supplementary Material S3: The Relationship between Age and Vaccine Hesitancy

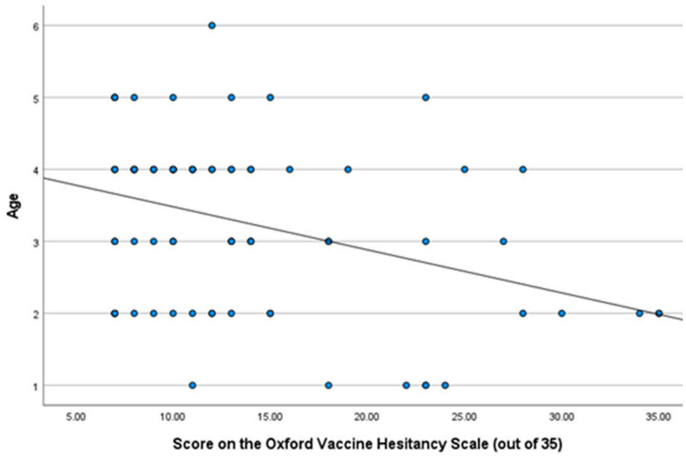

Figure 2. Scatter Graph showing the relationship between Age and Vaccine Hesitancy Scores out of 35 (A higher score indicating greater degree of hesitancy).

<u>**Supplementary Material S4 – Demographic Variables in the Sample.**</u>

<u>Table 2. Percentage and Frequency of Demographic Categories in the Sample. Fully vaccinated indicates receipt of 3 or more doses of the COVID-19 vaccine. (n=82).</u>

**Age Range (years)**	**N = (Percentage)**	**Percentage Fully Vaccinated**
18-25	6 (7.31)	50%
26-35	18 (21.9)	66.7%
36-45	16 (19.5)	81.3%
46-55	32 (39.1)	97%
56-65	8 (9.75)	100%
65+	1 (1.21)	100%
**Gender**	**N = (Percentage)**	**Percentage Fully Vaccinated**
Female	65 (79.3%)	71.6%
Male	17 (20.7%)	94.1%
**Ethnicity**	**N = (Percentage)**	**Percentage Fully Vaccinated**
White British	71 (86.6)	84.5%
Asian Indian	4 (4.87)	75%
White European	2 (2.43)	66.7%
Asian Filipino	1 (1.21)	100%
Black African	1 (1.21)	100%
Mauritian	1 (1.21)	100%
Arabic	1 (1.21)	100%
**Profession**	**N = (Percentage)**	**Percentage Fully Vaccinated**
Nurse	40 (48.7)	92.5%
Operating Department Practitioner	11 (13.41)	90%
Healthcare Assistant	10 (12.2)	40%
Pharmacist	8 (9.75)	100%
Doctor	7 (8.53)	75%
Care Worker	3 (3.65)	87.5%
Pharmacy Technician	2 (2.43)	50%
**Location**	**N = (Percentage)**	**Percentage Fully Vaccinated**
Channel Islands	46 (56.10)	89.2%
England	23 (28.0)	78.3%

Wales	12 (14.6)	75%
Isle Of Man	1 (1.21)	100%

Supplementary Material S4: Distribution of Scores in the Independent Variables

Whilst scores for BMQ-Necessity (Figure 1), BMQ-Concerns (Figure 2), BMQ-Harm (Figure 4) and BIPQ (Figure 7) were normally distributed, scores for BMQ-Overuse (Figure 5) and Vaccine Hesitancy (Figure 6) had a positive skew, meaning lower scores were more common for these measures. The distribution of Necessity-Concerns differentials had a negative skew, meaning higher scores (indicating higher necessity than concern) were more common. Descriptive statistics and Cronbach's alpha scores for the Independent Variables in the model can be found in Table S3.

Table 3. Descriptive Statistics and Cronbach's alpha scores of the Predictors used in the Model (n=82).

	**Min.**	**Max.**	**Mean**	**S.E.**	**Cronbach's α**
BMQ-Necessity	6.00	25.00	16.79 / 25.00	0.518	0.899
BMQ-Concerns	3.00	19.00	10.82 / 25.00	0.418	0.764
NCD	-13.00	20.00	5.93 / 25.00	7.112	N/A
BMQ-Harm	4.00	17.00	8.23 / 20.00	0.309	0.724
BMQ-Overuse	4.00	17.00	7.50 / 20.00	0.394	0.855
BIPQ	18.00	69.00	46.52 / 88.00	1.196	0.744
Vaccine Hesitancy	7.00	35.00	13.56 / 35.00	0.782	0.925

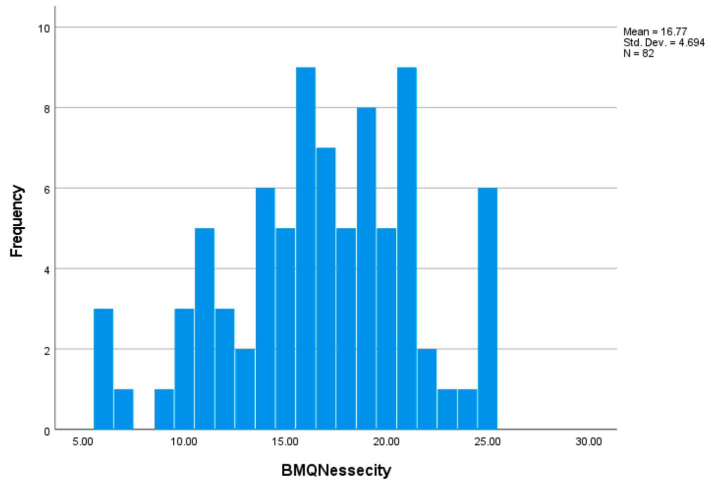

Figure 3. Histogram showing the distribution of BMQ-Necessity Scores

The range of BMQ-Necessity Scores was 6.00 - 25.00, with 59.8% being above the midpoint and 40.2% being below the midpoint.

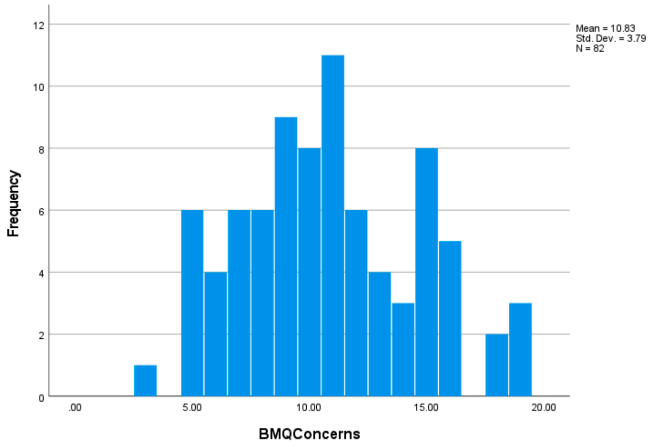

Figure 4. Histogram showing the distribution of BMQ-Concerns Scores

The range of BMQ-Concerns Scores was 3.00 - 19.00, with 45.1% being above the midpoint and 54.9% being below the midpoint.

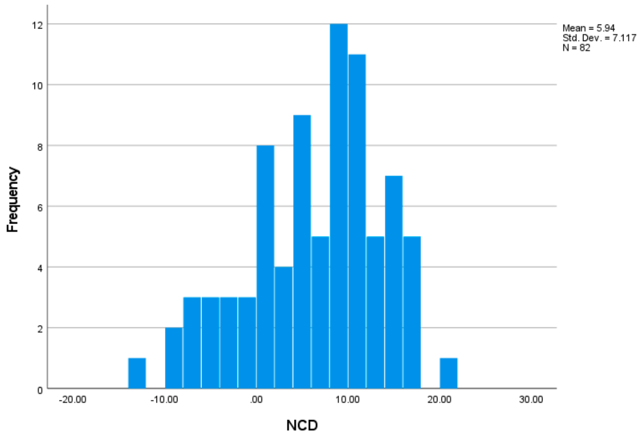

Figure 5. Histogram showing the distribution of Necessity-Concerns Differentials

The range of Necessity-Concerns Differentials was -13.00 - 20.00, with 58.5% being above the midpoint and 41.5% being below the midpoint.

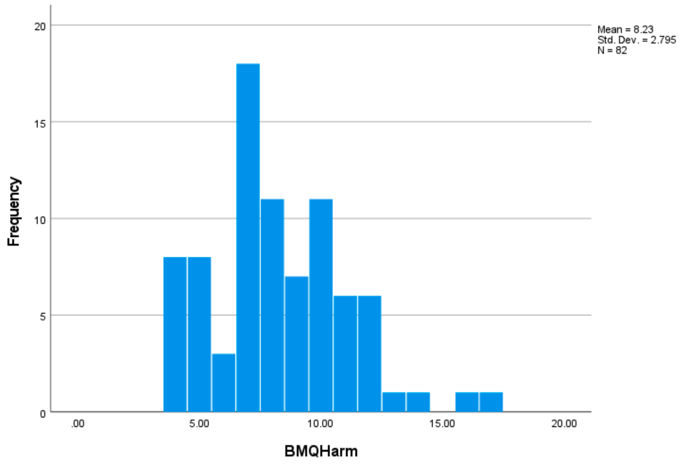

Figure 6. Histogram showing the distribution of BMQ-Harm Scores

The range of BMQ-Harm Scores was 4.00 - 17.00, with 13.4% being above the midpoint and 86.6% being below the midpoint.

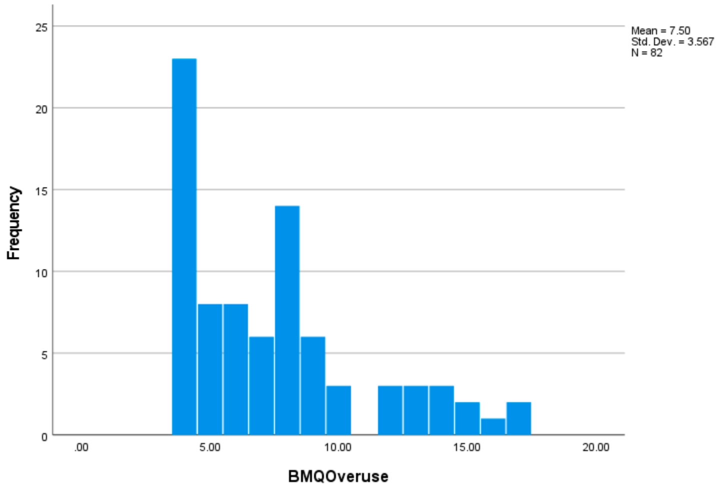

Figure 7. Histogram showing the distribution of BMQ-Overuse Scores

The range of BMQ-Overuse Scores was 4.00 - 17.00, with 17.1% being above the midpoint and 82.9% being below the midpoint.

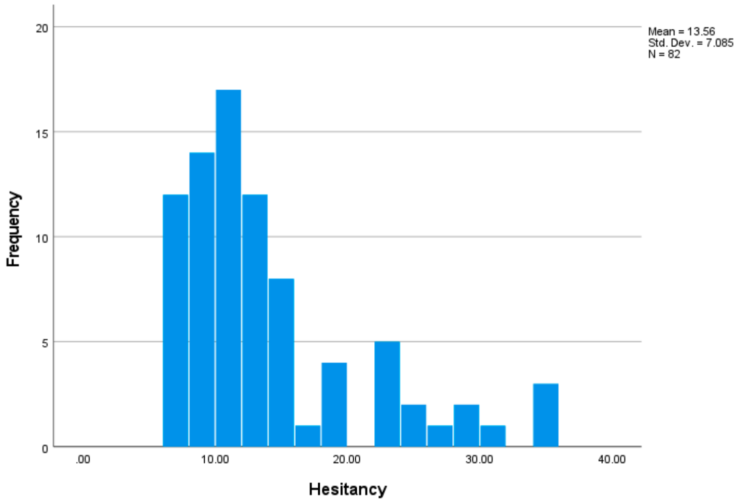

Figure 8. Histogram showing the distribution of Vaccine Hesitancy Scores

The range of Vaccine Hesitancy Scores was 7.00 - 35.00, with 17.1% being above the midpoint and 82.9% being below the midpoint.

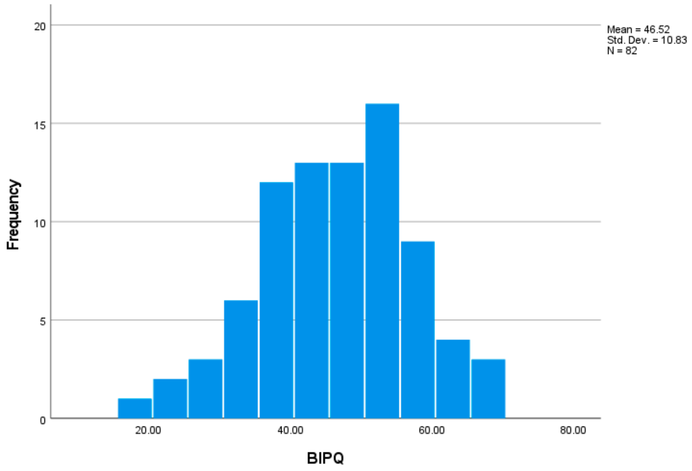

Figure 9. Histogram showing the distribution of BIPQ Scores

The range of BIPQ Scores was 18.00 - 69.00, with 39.0% being above the midpoint and 61.0% being below the midpoint.

Supplementary Material S5: Coefficients of predictors in the model from the Logistic Regression

A bivariate logistic regression was performed to assess the power of the model in predicting delay or no delay for all doses.

At Block 0 overall accuracy was 59.5% (0% for predicted "Delay" and 100% for predicted "No delay"). The model was not a significant contribution to predicting Delay (X^2=4.119, d.f. = 6, p = 0.661) accounting for between 5.4% and 7.3% of the variance in the DV (Cox & Snell R^2= 0.054., Nagelkerke R^2 = 0.073), see table of coefficients

Table S4. Coefficients of predictors in the model from the Logistic Regression

							95% C.I for EXP (B)	
	B	S.E	Wald	d.f.	Sig.	Exp(B)	Lower	Upper
BMQ-Necessity	-0.045	0.090	0.249	1	0.618	0.956	0.802	1.140
BMQ-Concerns	0.130	0.100	1.681	1	0.191	1.131	0.936	1.385
BMQ-Harm	-0.040	0.158	0.065	1	0.799	0.961	0.705	1.308
BMQ-Overuse	0.057	0.139	0.165	1	0.684	1.06	0.806	1.390
Hesitancy	-0.062	0.078	0.624	1	0.430	0.940	0.806	1.096
BIPQ	-0.032	0.028	1.361	1	0.243	0.968	0.917	1.022
NCD	-0.057	0.039	2.097	1	0.148	0.944	0.874	1.020

Supplementary Material S6: Quotations from Free-Text Responses

Table S5: Practical, Ambivalence, and Perceptual qualitative reasoning given for vaccine delay.

**Practical**	**Ambivalence**	**Perceptual**
"shift work + covid pos"	*"didn't think it was necessary"*	*"work, then unsure (didn't get 4th)"*
"contracted covid"	*"got it for travel"*	*"only took the vaccine whilst pregnant"*
"covid positive"		*"unsure whilst pregnant"*
"covid pos"		*"hesitant"*
"covid positive"		*"anti-vaxx friends causing doubt"*
"covid positive"		*"pregnancy"*
"covid pos"		
"covid positive"		
"COVID POS"		
"Couldn't book in"		
"short staffed"		
"Had Covid"		

Supplementary Material S7: Quantitative Questionnaire (Exported from www.qualtrics.com)

Survey 1: Quantitative Questionnaire

Start of Block: Demographics

Q1 Thank you for taking the time to fill in this survey. Please answer the following questions about yourself.

Page Break

Q2 How old are you?

- ○ 18-25
- ○ 26-35
- ○ 36-45
- ○ 46-55
- ○ 56-65
- ○ 66-75
- ○ 75+

Q3 Which gender do you identify with?

- ○ Male
- ○ Female
- ○ Other (Non-Binary, Agender)

Q4 What ethnicity would you describe yourself as?

- Black African
- Black Caribbean
- Asian Indian
- Asian Pakistani
- White British
- White European
- Chinese
- Other (Please State) ____

Q5 Did you work in the healthcare field during the COVID-19 pandemic?

- Yes
- No

Skip To: End of Survey If Did you work in the healthcare field during the COVID-19 pandemic? = No

---

Q6 In what healthcare profession did you work as during the COVID-19 pandemic?

- Nurse
- Doctor
- Healthcare Assistant
- Pharmacist
- Care/Support Worker
- Non-patient Facing Role
- Other (Please State) ____

Q7 Which country within the UK do you currently live in?

- England
- Wales
- Scotland
- Northern Ireland
- Other (please specify) ____

End of Block: Demographics

Start of Block: BMQ-S

Q4 *Your views about vaccines for yourself*

We would like to ask you about your personal views about accepting the COVID-19 vaccination.

These are statements other people have made about vaccination. Please indicate the extent to which you agree or disagree with them by ticking the box.

There are no right or wrong answers. We are interested in your personal views.

Q1 'My health, at present, depends upon being vaccinated against COVID-19'

- ○ Strongly agree
- ○ Agree
- ○ Neither agree nor disagree
- ○ Disagree
- ○ Strongly disagree

Q2 'Having to accept COVID-19 vaccinations worries me'

- ○ Strongly Agree
- ○ Agree
- ○ Neither agree nor disagree
- ○ Disagree
- ○ Strongly disagree

Q3 'My life would be impossible without being vaccinated against COVID-19'

- Strongly Agree
- Agree
- Neither agree nor disagree
- Disagree
- Strongly disagree

Q4 'Without COVID-19 vaccination I would become very ill'

- Strongly Agree
- Agree
- Neither agree nor disagree
- Disagree
- Strongly disagree

Q5 'I sometimes worry about the long-term effects of the COVID-19 vaccination'

- Strongly Agree
- Agree
- Neither agree nor disagree
- Disagree
- Strongly disagree

Q6 'COVID-19 Vaccination is a mystery to me'

- ○ Strongly Agree
- ○ Agree
- ○ Neither agree nor disagree
- ○ Disagree
- ○ Strongly disagree

Q7 'My health in the future will depend upon being vaccinated against COVID-19'

- ○ Strongly Agree
- ○ Agree
- ○ Neither agree nor disagree
- ○ Disagree
- ○ Strongly disagree

Q8 'COVID-19 vaccination has disrupted my life'

- ○ Strongly Agree
- ○ Agree
- ○ Neither agree nor disagree
- ○ Disagree
- ○ Strongly disagree

Q9 'I sometimes worry about becoming too dependent on the COVID-19 vaccinations'

◯ Strongly Agree

◯ Agree

◯ Neither agree nor disagree

◯ Disagree

◯ Strongly disagree

Q10 'Vaccination against COVID-19 protects me'

◯ Strongly Agree

◯ Agree

◯ Neither agree nor disagree

◯ Disagree

◯ Strongly disagree

End of Block: BMQ-S

Start of Block: BMQG

Q14 Your views about vaccinations in general

We would like to ask you about your personal views about vaccinations in general.

These are statements other people have made about medicines in general. Please indicate the extent to which you agree or disagree with them by ticking the appropriate box.

There are no right or wrong answers. We are interested in your personal views.

Q11 Vaccinations are recommended too freely'

- Strongly Agree
- Agree
- Neither agree nor disagree
- Disagree
- Strongly disagree

Q12 'People should think hard about whether they want to become vaccinated'

- Strongly Agree
- Agree
- Neither agree nor disagree
- Disagree
- Strongly disagree

Q13 'Vaccination is not necessary'

- Strongly Agree
- Agree
- Neither agree nor disagree
- Disagree
- Strongly disagree

Q14 'Natural remedies are safer than vaccinations'

- Strongly Agree
- Agree
- Neither agree nor disagree
- Disagree
- Strongly disagree

Q15 'Vaccination does more harm than good'

- Strongly Agree
- Agree
- Neither agree nor disagree
- Disagree
- Strongly disagree

Q16 'Vaccinations are poisons'

- Strongly Agree
- Agree
- Neither agree nor disagree
- Disagree
- Strongly disagree

Q17 'Doctors place too much trust in vaccinations'

- Strongly Agree
- Agree
- Neither agree nor disagree
- Disagree
- Strongly disagree

Q18 'If doctors spent more time with patients they would be less likely to recommend vaccinations'

- Strongly Agree
- Agree
- Neither agree nor disagree
- Disagree
- Strongly disagree

End of Block: BMQG

Start of Block: BIPQ

Q1 For the following questions, please select the number that best corresponds to your views:

	0 - no affect at all	1	2	3	4	5	6	7	8	9	10 - would severely affect my life
How much would catching COVID-19 affect your life?	○	○	○	○	○	○	○	○	○	○	○

Q2

	0 - a very short time	1	2	3	4	5	6	7	8	9	10 - forever
How long do you think the effects of catching COVID-19 would last?	○	○	○	○	○	○	○	○	○	○	○

Q3

	0 - no control at all	1	2	3	4	5	6	7	8	9	10 - extreme control
How much control do you feel over whether you catch COVID-19?	○	○	○	○	○	○	○	○	○	○	○

Q4

	0 - not at all	1	2	3	4	5	6	7	8	9	10 - extremely helpful
How much do you think that treatment can help with symptoms of COVID-19?	○	○	○	○	○	○	○	○	○	○	○

Q4

	0 - no symptoms at all	1	2	3	4	5	6	7	8	9	10 - many severe symptoms
How severe do you think the symptoms of COVID-19 are?	○	○	○	○	○	○	○	○	○	○	○

Q5

	0 - not at all concerned	1	2	3	4	5	6	7	8	9	10 - extremely concerned
How concerned are you about COVID-19?	○	○	○	○	○	○	○	○	○	○	○

Q6

	0 - don't understand at all	1	2	3	4	5	6	7	8	9	10 - understand very clearly
How well do you feel you understand COVID-19?	○	○	○	○	○	○	○	○	○	○	○

Q7

	0 - not at all affected?	1	2	3	4	5	6	7	8	9	10 - extremely affected
How much does COVID-19 affect you emotionally?	○	○	○	○	○	○	○	○	○	○	○

End of Block: BIPQ

Start of Block: VHS

Q1 If another COVID-19 vaccine booster was made available, would you take it?

○ Definitely

○ Probably

○ I may or may not

○ Probably not

○ Definitely not

○ I don't know

Q2 If another COVID-19 vaccine booster was made available:

- I will want to get it as soon as possible
- I will take it when offered
- I'm not sure what I will do
- I will delay getting in
- I will refuse to get it
- I don't know

Q3 When the COVID-19 vaccine first became available (in January 2021), I would describe my attitude towards it as:

- Very keen
- Pretty positive
- Neutral
- Quite uneasy
- Against it
- I don't know

Q4 If another COVID-19 booster vaccine was available at my local pharmacy, I would:

- Get it as soon as possible
- Get it when I have time
- Delay getting it
- Avoid getting it for as long as possible
- Never get it
- I don't know

Q5 If my family or friends were thinking of getting a COVID-19 vaccination, I would:

- Strongly encourage them
- Encourage them
- Not say anything to them about it
- Ask them to delay getting the vaccination
- Suggest that they don't get the vaccination
- I don't know

Q6 I would describe myself as:

- Strongly favorable towards the COVID vaccine
- Favorable towards the vaccine
- Neutral towards the vaccine
- Hesitant towards the vaccine
- Anti-vaccination for the COVID vaccine
- I don't know

Q7 Accepting a COVID vaccination is:

- Really important
- Important
- Neither important nor unimportant
- Unimportant
- Really unimportant
- I don't know

End of Block: VHS

Start of Block: NM

Q1 How many doses of the COVID-19 vaccination have you currently received?

- 0
- 1
- 2
- 3
- 4

Q2 We are interested in finding out if you delayed receiving the COVID-19 vaccination for any reason. For each dose, type the date of the appointment and the date you received the vaccine. If you have not been vaccinated, please put Not Applicable for each dose

○ First Dose - Month of appointment given: (N/A if not vaccinated)

____

○ First Dose - How many weeks following this appointment did you receive the vaccine? (0 if received vaccine on first date given)

____

○ Reason for delay, if any: ____

○ Second dose - Month of appointment given (N/A if not vaccinated):

____

○ Second dose - How many weeks following this appointment did you receive the vaccine? (0 if received vaccine on first date given)

____

○ Reason for delay, if any: ____

○ Third dose - Month of appointment given (N/A if not vaccinated):

____

○ Third dose - How many weeks following this appointment did you receive the vaccine? (0 if received vaccine on first date given)

____

○ Reason for delay, if any: ____

○ Fourth Dose Offered? ____

○ Month of appointment: ____

○ How many weeks following this appointment given did you receive the vaccine? (0 if received vaccine on first date given)

____

○ Reason for delay (if any):

____

End of Block: NM

Start of Block: Block 8
